# Population-Level Effects of Human Papillomavirus Vaccination Programs on Infections with Nonvaccine Genotypes

**DOI:** 10.3201/eid2210.160675

**Published:** 2016-10

**Authors:** David Mesher, Kate Soldan, Matti Lehtinen, Simon Beddows, Marc Brisson, Julia M.L. Brotherton, Eric P.F. Chow, Teresa Cummings, Mélanie Drolet, Christopher K. Fairley, Suzanne M. Garland, Jessica A. Kahn, Kimberley Kavanagh, Lauri Markowitz, Kevin G. Pollock, Anna Söderlund-Strand, Pam Sonnenberg, Sepehr N. Tabrizi, Clare Tanton, Elizabeth Unger, Sara L. Thomas

**Affiliations:** Public Health England, London, UK (D. Mesher, K. Soldan, S. Beddows);; London School of Hygiene and Tropical Medicine, London (D. Mesher, S.L. Thomas);; University of Tampere, Tampere, Finland (M. Lehtinen); Imperial College London, London (M. Brisson);; Centre de recherche du CHU de Québec, Quebec City, Quebec, Canada (M. Brisson, M. Drolet);; Université Laval, Quebec (M. Brisson, M Drolet);; Victorian Cytology Service, Melbourne, Victoria, Australia (J.M.L. Brotherton);; University of Melbourne, Melbourne (J.M.L. Brotherton, S.M. Garland, S.N. Tabrizi);; Melbourne Sexual Health Centre, Melbourne (E.P.F. Chow, C.K. Fairley);; Monash University, Melbourne (E.P.F. Chow, C.K. Fairley);; Indiana University School of Medicine, Indianapolis, Indiana, USA (T. Cummings);; Murdoch Childrens Research Institute, Parkville, Victoria, Australia (S.M. Garland, S.N. Tabrizi);; The Royal Women’s Hospital, Melbourne (S.M. Garland, S.N. Tabrizi);; Cincinnati Children’s Hospital Medical Center, Cincinnati, Ohio, USA (J.A. Kahn);; University of Cincinnati College of Medicine, Cincinnati (J.A. Kahn);; University of Strathclyde, Glasgow, Scotland, UK (K. Kavanagh);; Centers for Disease Control and Prevention, Atlanta, Georgia, USA (L. Markowitz, E. Unger);; Health Protection Scotland, Glasgow (K.G. Pollock);; Division of Clinical Microbiology, Laboratory Medicine, Skåne, Lund, Sweden (A. Söderlund-Strand);; University College London, London (P. Sonnenberg, C. Tanton)

**Keywords:** human papillomavirus, HPV, HPV vaccination, surveillance, nonvaccine types, viruses, vaccines, genotypes

## Abstract

We analyzed human papillomavirus (HPV) prevalences during prevaccination and postvaccination periods to consider possible changes in nonvaccine HPV genotypes after introduction of vaccines that confer protection against 2 high-risk types, HPV16 and HPV18. Our meta-analysis included 9 studies with data for 13,886 girls and women ≤19 years of age and 23,340 women 20–24 years of age. We found evidence of cross-protection for HPV31 among the younger age group after vaccine introduction but little evidence for reductions of HPV33 and HPV45. For the group this same age group, we also found slight increases in 2 nonvaccine high-risk HPV types (HPV39 and HPV52) and in 2 possible high-risk types (HPV53 and HPV73). However, results between age groups and vaccines used were inconsistent, and the increases had possible alternative explanations; consequently, these data provided no clear evidence for type replacement. Continued monitoring of these HPV genotypes is important.

## Abstract 2

After introduction of vaccination, some prevalences of nonvaccine types changed, without clear evidence for type replacement.

Persistent infection with a high-risk human papillomavirus (HPV) genotype is necessary for development of cervical cancer (*1*). Two high-risk types, HPV16 and HPV18, cause ≈70%–80% of cervical cancers (*2**–**4*). The HPV vaccines currently available commercially have been shown in trial settings to have ≈100% vaccine efficacy against cervical disease caused by vaccine-specific high-risk HPV types: bivalent and quadrivalent vaccines against HPV16 and HPV18 and the new nonavalent vaccine against HPV31, HPV33, HPV45, HPV52, and HPV58 (*5**–**7*). Clinical trial data for the bivalent and quadrivalent vaccines have shown low-to-moderate protection (i.e., cross-protection) against other high-risk HPV types that are phylogenetically related to HPV16 and HPV18 (*8**,**9*).

Many countries have now introduced HPV vaccination programs (*10*). A recently published systematic review and meta-analysis assessed population-level effects of HPV vaccination on vaccine HPV types and showed strong evidence that HPV vaccination is highly effective against infections with these vaccine-specific high-risk types (*11*). The review also examined closely related HPV types as a single group and found evidence of cross-protection overall in a population-based setting (*11*). However, assessment of changes in the prevalence of closely related HPV types combined may not provide full evidence of the effects of a national vaccination program because examining the types as a single group potentially conceals decreases or increases in the prevalence of individual types. Grouping HPV types together limits the possibility of examining cross-protection provided by specific HPV types and of detecting changes in other individual nonvaccine types. For example, a theoretical concern is that reduced prevalences of infection with HPV16 and HPV18 could lead to other high-risk HPV types occupying those niches and becoming more common causes of disease. Although type replacement was not observed in the clinical trials (*12*), monitoring for possible type replacement in population-based settings after the introduction of national HPV vaccination programs is important. Furthermore, because nonvaccine HPV types are far less common than vaccine HPV types, a single study may have limited scope to determine whether type replacement has occurred. Combining data from several reports improves the ability to investigate type replacement. We aimed to investigate population-level effects of HPV vaccination programs that used bivalent or quadrivalent vaccines on type-specific prevalences of infection caused by individual nonvaccine high-risk HPV types.

## Methods

### Objectives

Using data from surveys conducted before an HPV vaccination program was introduced and data from surveys after the program was introduced, we compared HPV prevalences for similar populations within the same country. We conducted a systematic literature search to determine changes in HPV prevalence for each nonvaccine high-risk HPV type. At the time of our search, any eligible study would have considered vaccination that used bivalent or quadrivalent vaccines; consequently, high-risk HPV types used only in the nonavalent vaccine were considered nonvaccine HPV types. Each individual type was presented separately in our analysis. We included HPV types for which some cross-protection had been demonstrated in clinical trials (HPV31 and HPV33, which are phylogenetically related to HPV16, and HPV45, which is phylogenetically related to HPV18) (*8**,**9**,**13*); other high-risk HPV types included in the nonavalent vaccine (HPV52 and HPV58); other high-risk and probably high-risk HPV types (HPV35, HPV39, HPV51, HPV56, HPV59, and HPV68); and other possibly high-risk HPV types (HPV26, HPV53, HPV70, HPV73, and HPV82), as classified by the International Agency for Research on Cancer (*14*). This systematic review and meta-analysis was reported in accordance with PRISMA guidelines (*15*).

### Search Strategy and Selection Criteria

Using Embase, Medline, LILACS, and African Index Medicus databases, we searched for eligible publications published from 2007, the year that the first HPV vaccination programs were introduced, through February 19, 2016. To identify relevant studies that mentioned both vaccination and HPV infection or a related disease (such as HPV-related precancerous lesions, cancers, and genital warts), the search strategy incorporated MeSH terms from the PubMed database (http://www.ncbi.nlm.nih.gov/mesh) and relevant words found in the title or abstract (Technical Appendix). The search had no language restrictions.

Eligible studies were those that assessed population-level effects of HPV vaccination over time by comparing the prevalence of HPV infection (defined by the detection of HPV DNA in patient samples) during a prevaccination period with the prevalence during a postvaccination period. We excluded studies comparing HPV infection in vaccinated persons with HPV infection in unvaccinated persons as part of an individually randomized trial because such studies would not measure population-level effects. Similarly, we excluded studies in which HPV infection was compared only between unvaccinated and vaccinated persons in the postvaccination period. We also excluded studies in which only a small proportion (<2%) of the postvaccination study population was vaccinated (i.e., studies conducted in largely unvaccinated populations). One author (D.M.) initially reviewed titles and abstracts of studies for eligibility; we reviewed in full those studies that appeared to address changes in HPV prevalence after introduction of HPV vaccination programs. We also compared search results with those identified in a recent related review (*11*), which compared prevaccination and postvaccination periods for high-risk vaccine types (HPV16 and HPV18), cross-protected types (HPV31, HPV33, and HPV45), and all high-risk HPV nonvaccine types combined.

### Data Extraction and Data Quality

For each study, we extracted data on study design and country of study. Then, for both prevaccincation and postvaccination periods, we extracted data on year(s) of sample collection, study setting and population, sample size, specimen type, assay used for HPV DNA testing, HPV genotypes included in the assay, demographic and sexual behavior data collected, and the measure of effect (and method used to determine any effect). For the postvaccination period, we also extracted data on the method used to ascertain estimated vaccination coverage.

In addition, we assessed the potential bias in each study by considering the comparability of the study populations in the prevaccination versus postvaccination periods (i.e., similar setting and population demographics); the extent of adjustment for potential confounders; the suitability of the specimen type to assess HPV DNA infection; the suitability of the assay used for accurate HPV DNA testing (and whether the suitability of assays differed between the prevaccination and postvaccination periods); and the method used to estimate HPV vaccination coverage. To assess external validity, we considered whether the study samples were population based. Each of these factors was scored as either low risk or high risk.

When published data on HPV prevalence and prevalence ratios (PRs) for individual high-risk HPV types were unavailable, we contacted authors to request the HPV type-specific prevalences during the prevaccination and postvaccination periods and the PRs for the 2 periods for each nonvaccine high-risk HPV type. We requested PRs adjusted for demographic and sexual behavior data or the unadjusted PRs if data on confounders were unavailable; we calculated unadjusted PRs if authors provided raw data. By using data from a previously conducted validation study, 1 study included adjusted odds ratios rather than PRs to adjust for the change in assay used during the prevaccination and postvaccination periods (*16*).

### Data Analysis

We used estimates weighted to account for selection processes if that data were available from authors (unweighted numbers are shown in Technical Appendix Table 1). We also stratified data by age group (i.e., <19 and 20–24 years of age) because of expected lower rates of vaccination coverage and lower vaccine effectiveness in those vaccinated at older ages. Consequently, for each study, we requested data from authors for the same 2 age groups. One study included data for girls <13 years of age, so we requested data restricted to those 16–19 years of age (*17*).

To enable calculation of a PR for a prevalence of 0 during either the prevaccination or postvaccination period, we used a continuity correction of 0.5. When prevalence was 0 for both the prevaccination and postvaccination periods, we excluded the study from the meta-analysis for the relevant age group and HPV type. Results were further stratified by type of vaccine used (i.e., bivalent or quadrivalent). PRs within each subgroup were combined to obtain a summary PR by using a fixed-effects model if data were not shown to be heterogeneous; lack of heterogeneity was determined by a p value >0.10 calculated with the Cochrane Q test or by an I^2^ value <25% (*18*)*.* Sensitivity analyses were restricted to studies that used cervical, vulval, or vaginal swabs as specimen type because urine samples have lower sensitivity for detecting HPV DNA infection (*19*).

## Results

### Included Studies

**Figure 1:**
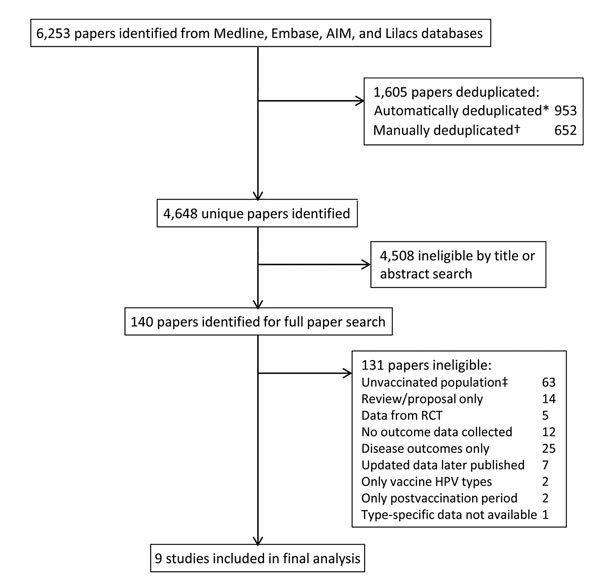
Flowchart for eligible studies included in systematic review and meta-analysis of changes in prevalences of nonvaccine human papillomavirus (HPV) genotypes after introduction of HPV vaccination. *100% title match, author’s surname and initial, publication year, and periodical; 85% title match, and author surname; ‡includes studies in which the vast majority of the population were unvaccinated. RCT, randomized controlled trials.

After we eliminated duplications, we identified 4,648 unique articles in searches from all 4 databases (Figure 1). An initial search of title and abstracts of these articles excluded 4,508 (97.0%) because of ineligibility. For the remaining 140 articles, we examined the full text to determine compliance with eligibility criteria and identified 10 eligible studies (Figure 1). Of these 10 studies, 1 met all eligibility criteria, but the type-specific PRs were unavailable from authors (*20*). Therefore, we included 9 studies in the systematic review and meta-analysis (*16**,**17**,**21**–**27*). All eligible studies were repeat cross-sectional studies that compared changes in prevalence in populations before and after introduction of a national HPV vaccination program (Technical Appendix Table 1). Because only 1 study considered changes in HPV infection among male and female populations, we considered only female populations in the analysis. Two studies were population-based national surveys (*23**,**26*); 3 studies were conducted among young women obtaining chlamydia screening (*16**,**17**,**27*); 2 studies comprised young women attending a primary care clinic, community health center, or hospital-based adolescent clinic (*21**,**22*); and 2 studies comprised women obtaining cervical screening (*24**,**25*) (Technical Appendix Table 1). The included studies contained data on 13,886 girls and women <19 years of age and 23,340 women 20–24 years of age.

The studies varied in methodologic quality on the basis of potential bias (Table 1). Most studies collected some demographic and sexual behavior data to enable appropriate adjustment of the relative risks, although the number of factors collected was limited in some studies (*16**,**17**,**24**,**25*) (Table 1; Technical Appendix Table 1). 

**Table 1:** Potential bias and external validity of studies included in meta-analysis of changes in prevalences of nonvaccine HPV genotypes*

Potential bias factors	Study, authors (reference no.)								
	Mesher et al. (*16*)	Söderlund-Strand et al. (*1**7*)	Cummings et al. (*21*)	Kahn et al. (*22*)	Sonnenberg et al. (*23*)	Tabrizi et al. (*24*)	Cameron et al. (*25*)	Markowitz et al. (*26*)	Chow et al. (*27*)
Population-based samples†	**H**	**H**	**H**	**H**	L	L	L	L	**H**
Comparative populations†	**H**	**H**	L	L	L	L	L	L	**H**
Risk factor data collected and adjusted for	**H**	**H**	L	L	L	**H**	**H**	L	L
Samples suitable for assessing HPV	L	L	L	L	**H**	L	L	L	L
Assay with suitable accuracy	L	L	L	L	L	L	L	L	L
Identical HPV assays†	**H**	L	L	L	L	L	L	L	L
Vaccination status collected	**H**	**H**	L	L	**H**	L	L	**H**	**H**

*HPV, human papillomavirus; H (in bold), high risk of bias; L, low risk of bias. †For both prevaccination and postvaccination periods.

### HPV Types Included in Nonavalent HPV Vaccines 

#### HPV Types with Prior Evidence for Cross-Protection 

**Figure 2:**
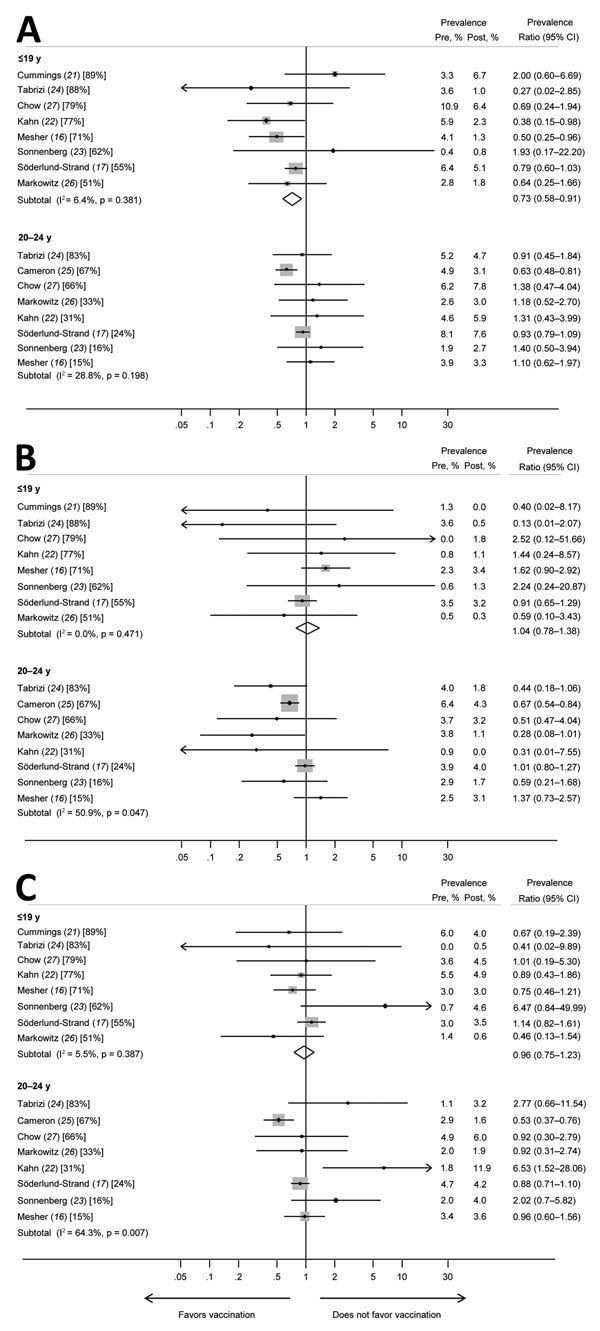
Prevalence ratios and 95% CIs for high-risk human papillomavirus (HPV) types (HPV31, HPV33, and HPV45) that had evidence of cross-protection for girls and women <19 years of age and women 20–24 years of age in studies included in a meta-analysis of changes in prevalences of nonvaccine HPV genotypes after introduction of HPV vaccination. A) HPV31; B) HPV33; C) HPV45. Percentages in brackets represent vaccination coverage (>1 dose) for each study and age group. The size of the gray boxes around the plot points indicates the relative weight given to each study in the calculation of the summary estimate. The study by Cameron et al. (*25*) is omitted from analyses for the younger age group because this study included no data for the group <19 years of age. The study by Cummings et al. (*21**)* is omitted from analyses for women 20–24 years of age because this study included no data for this age group. Pre, prevaccination; post, postvaccination.

We found evidence of reduced prevalence of HPV31 (Figure 2; Table 2) among girls and women <19 years of age (PR 0.73, 95% CI 0.58–0.91) but found little evidence of changed prevalences for HPV33 or HPV45 among this age group (PR 1.04, 95% CI 0.78–1.38 for HPV33; PR 0.96, 95% CI 0.75–1.23 for HPV45). Results were heterogeneous for HPV31, HPV33, and HPV45 in women 20–24 years of age; consequently, we did not calculate summary PRs (Figure 2; Table 2).

**Table 2:** Summary prevalence ratios for meta-analysis of changes in nonvaccine high-risk HPV types among girls and women, by age group*

Population age group, y, and HPVtype	No. studies†	Heterogeneity		Prevalence ratio (95% CI)
		I^2^, %	p value	
≤19				
HPV types in nonavalent vaccine	8			
HPV31		6.4	0.381	0.73 (0.58–0.91)
HPV33		0	0.471	1.04 (0.78–1.38)
HPV45		5.5	0.387	0.96 (0.75–1.23)
HPV52		24.0	0.238	1.34 (1.13–1.59)
HPV58		0	0.727	1.01 (0.80–1.26)
Other high-risk HPV types	8			
HPV35		25.1	0.229	–
HPV39		0	0.984	1.27 (1.05–1.54)
HPV51		43.6	0.088	–
HPV56		74.3	<0.001	–
HPV59		66.8	0.004	–
HPV68		0	0.690	1.26 (0.88–1.81)
Other possibly high-risk HPV types	6			
HPV26		0	0.478	1.63 (0.84–3.16)
HPV53		3.6	0.394	1.51 (1.10–2.06)
HPV70		23.6	0.257	1.34 (0.75–2.39)
HPV73		0	0.961	1.36 (1.03–1.80)
HPV82		49.0	0.081	–
20–24				
HPV types in nonavalent vaccine	8			
HPV31		28.8	0.198	–
HPV33		50.9	0.047	–
HPV45		64.3	0.007	–
HPV52		31.0	0.180	–
HPV58		0	0.806	1.14 (0.99–1.31)
Other high-risk HPV types	8			
HPV35		7.9	0.369	1.07 (0.85–1.34)
HPV39		0	0.522	1.13 (1.00–1.28)
HPV51		49.8	0.052	–
HPV56		82.6	<0.001	–
HPV59		63.6	0.007	–
HPV68		35.6	0.145	–
Other possibly high-risk HPV types	6			
HPV26		44.3	0.110	–
HPV53		30.8	0.204	–
HPV70		25.1	0.246	–
HPV73		59.2	0.032	–
HPV82		38.3	0.151	–

*HPV, human papillomavirus; –, prevalence ratio not calculated because of heterogeneity of data. †Number of studies was the same for all HPV types within each category.

#### Other HPV Types 

**Figure 3:**
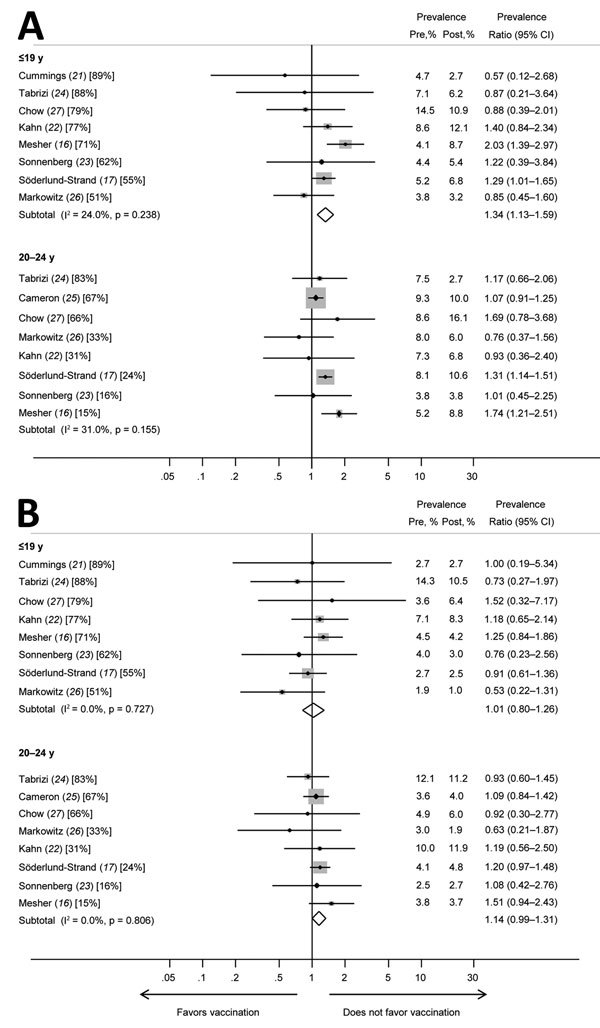
Prevalence ratios and 95% CIs for other high-risk human papillomavirus (HPV) types (HPV52 and HPV58) included in the nonavalent vaccine for girls and women <19 years of age and women 20–24 years of age in studies included in a meta-analysis of changes in prevalences of nonvaccine HPV genotypes after introduction of HPV vaccination. A) HPV52; B) HPV58. Percentages in brackets represent vaccination coverage (>1 dose) for each study and age group. The sizes of the gray boxes around the plot points indicates the relative weight given to each study in the calculation of the summary estimate. The study by Cameron et al. (*25*) is omitted from analyses for the younger age group because this study included no data for persons <19 years of age. The study by Cummings et al. (*21**)* is omitted from analyses for women 20–24 years of age because the study included no data for this age group. Pre, prevaccination; post, postvaccination.

We found evidence of increased prevalence of HPV52 in those <19 years of age (PR 1.34, 95% CI 1.13–1.59) (Figure 3; Table 2), but because of heterogeneity, we did not calculate summary PRs for those 20–24 years of age. We found no evidence of a changed prevalence for HPV58 among the younger age group (PR 1.01, 95% CI 0.80–1.26) but found borderline evidence of an increase for those 20–24 years of age (PR 1.14, 95% CI 0.99–1.31).

### Other High-Risk and Possibly High-Risk HPV Types

No consistent patterns appeared across the studies for other HPV vaccine types not used in the nonavalent vaccine (Table 2; Technical Appendix Figure 1). We found evidence of increased prevalences from the prevaccination period to the postvaccination period in those <19 years of age for HPV39 (PR 1.27, 95% CI 1.05–1.54), HPV53 (PR 1.51, 95% CI 1.10–2.06), and HPV73 (PR 1.36, 95% CI 1.03–1.80). For women 20–24 years of age, evidence indicated increased prevalence for HPV39 (PR 1.13, 95% CI 1.00–1.28).

### Sensitivity Analysis

As a sensitivity analysis, we performed 3 additional analyses, all stratified by age group: by type of vaccine used (i.e., bivalent or quadrivalent); by potential bias of the original study (i.e., relatively low potential bias, defined as <3 factors indicating high risk of bias; or relatively high potential bias, defined >3 factors indicating high risk of bias) (Table 1); and by vaccination coverage (i.e., low <50%; high >50%). For studies in settings that used the bivalent vaccine, we found evidence of increased prevalence between the prevaccination period and postvaccination periods among those <19 years of age for HPV52, HPV53, HPV56, and HPV70 (Technical Appendix Table 2, Figures 2–4). Prevalence of HPV53 among women 20–24 years of age also increased. For the quadrivalent vaccine, evidence showed increased prevalences of HPV39, HPV51, and HPV59 for those <19 years of age. Among those 20–24 years of age, evidence indicated increased prevalence of HPV52 and HPV70 (Technical Appendix Table 2, Figures 2–4).

Many of our analyses that were stratified by potential bias of the included studies had results similar to those in the unstratified analyses (Technical Appendix Table 3). However, among those <19 years of age, studies with a relatively low potential bias showed no evidence of increased prevalence for HPV52 or HPV39, although evidence existed when the studies were unstratified. For studies with relatively high potential bias, among this younger age group, evidence showed increased prevalences of HPV51 and HPV70, although these increases were not present in the unstratified analysis. In women 20–24 years of age, evidence showed decreased prevalence for HPV33 in those studies with a relatively low potential bias. No summary estimate was provided in the unstratified analysis because of heterogeneity of data. Studies with a relatively high potential bias showed evidence of increased prevalences of HPV52 and HPV58 among women 20–24 years of age. Among this older age group, evidence existed for decreased prevalence of HPV82 in those studies with both relatively high potential bias and relatively low potential bias, although those studies with relatively high potential bias had a larger decrease. Again, no summary estimate was provided in the unstratified analysis because of heterogeneity.

Vaccination coverage was high for the younger age group in all studies (Technical Appendix Table 4). For the older age group, studies with high vaccination coverage showed decreased prevalence for HPV31. No summary estimate was provided for the unstratified analysis because of heterogeneity. For the older age group, we found evidence of increased prevalences for HPV39 and HPV58 (similar to results from the unstratified analysis) but only in studies with low vaccination coverage. Although not seen in the unstratified analysis, we also found evidence of an increased prevalence for HPV70 in low-coverage studies and borderline evidence of an increased prevalence for HPV26 in high-coverage studies. No summary estimates were provided for the unstratified analyses because of heterogeneity.

## Discussion

Comprehensive postvaccination surveillance should not only consider reductions of vaccine type–specific infection and associated disease but should also assess any other potential effects of reductions of targeted infections. We assessed changes in nonvaccine HPV types to determine evidence of cross-protection for individual HPV types and to investigate the potential concern that reductions in certain HPV types after the introduction of HPV vaccination in a population could create a niche that enables other nonvaccine high-risk HPV types to become more common (i.e., type replacement). We found evidence of a reduction in the prevalence of HPV31 among girls and women <19 years of age. Our main analysis showed increases in other nonvaccine HPV types (HPV39, HPV52, HPV53, HPV58, and HPV73), but these increases were inconsistent for the 2 age groups examined and the vaccines used.

A previous systematic review evaluated changes in high-risk HPV types combined and found evidence of a reduction in the prevalence of HPV types closely related to vaccine types (HPV31, HPV33, and HPV45) when they were considered as a single group (PR 0.72, 95% CI 0.54–0.96 for girls and women 13–19 years of age) (*11*). Our review provides evidence of reduced prevalence for HPV31 but little evidence of reduced prevalence for HPV33 or HPV45.

Comparing HPV prevalence in a prevaccination period to prevalence in a similar population in a postvaccination period enables consideration of population-level effects of HPV vaccination on HPV prevalence. However, these repeat cross-sectional study designs have limitations. Although all studies addressed similar populations in the prevaccination and postvaccination periods, these populations may have undergone temporal changes that are independent of HPV vaccination over time and that possibly affect HPV prevalence. For example, increases in diagnoses of other sexually transmitted infections have occurred during the same period as that of HPV vaccination programs (*28*). Furthermore, incidence of genital warts increased in many countries before vaccine introduction (*29**–**31*) and has continued to increase postvaccination in persons ineligible for vaccination (*11*). Such findings suggest that the increases we observed in some HPV types are possibly associated with broad increases in sexual risk over time. We considered changes in demographics and sexual behavior for the populations over time when information was available, but unrecorded population changes or other temporal changes affecting the relative proportions of high-risk HPV types likely occurred over time (*32**,**33*). Also, more geographic variation exists in the relative frequency of nonvaccine HPV types in populations compared with the prevalence of HPV16, which, before the vaccination programs, was the most frequent high-risk type observed in almost all populations (*34*).

Furthermore, the change in assay used during the prevaccination and postvaccination periods was a potential source of bias in 1 study (*16*), which calculated odds ratios (ORs) adjusted for differences in diagnostic accuracy. This adjusted OR could not be converted to a PR by using the log-binomial model and was included as an OR. However, given the low prevalence of individual HPV types, the use of an OR instead of a PR for this study was unlikely to have affected the results substantially.

Another limitation is that the broad-spectrum assays used in these studies (and in baseline prevaccination evaluations globally) can lack sensitivity for detecting individual HPV types when multiple types are present, particularly if another HPV type with a higher viral load is present. In the postvaccination period, in the absence of HPV16 and HPV18, this lack of sensitivity could lead to an apparent but artificial increase in nonvaccine types because these types were underestimated in the prevaccine period because of the predominance of HPV16 or HPV18. Studies have shown this potential unmasking effect (*35**,**36*); some increases in nonvaccine types that we observed could result from unmasking.

Given the low prevalence of some nonvaccine HPV types, assessing changes in prevalence for individual types since the introduction of HPV vaccination has been challenging. By combining data from several studies, we enhanced our power to consider changes in individual HPV types. However, even with data from 13,886 girls and women <19 years of age and 23,340 women 20–24 years of age, we still had limited power to consider changes in very rare HPV types or to investigate reasons for the heterogeneity in findings for some HPV types because of inconsistent evidence for increases of specific nonvaccine types between age groups and the 2 (i.e., bivalent and quadrivalent) vaccines. Conversely, type 1 errors can occur with multiple testing, so modest evidence for increases should be interpreted with caution.

We decided against performing random-effects meta-analyses in the presence of between-study heterogeneity because, in most instances, inconsistency occurred in the direction of effect, making a summary estimate (i.e., the average value of these opposing effects) uninformative (*37*). Exploring the causes of heterogeneity could provide further insight into the reasons for these increases, so we performed 3 subgroup analyses by vaccine used, potential bias, and vaccine coverage. Results of the stratification by potential bias suggested that increased PRs for some HPV types may have been reported more often in the studies with relatively high potential bias. However, for all 3 subgroup sensitivity analyses, the small number of studies in each stratum limited the interpretation of the analyses. Similarly, we were limited to only 8 studies for each age group and had insufficient ability to perform meta-regression analyses (because meta-regression should generally not be considered for <10 studies) (*37*). As further data accrue, a useful future analysis would be exploring the association between reductions in the HPV vaccine types and any increases (not resulting from unmasking) in nonvaccine HPV types. If increases result from type replacement, then we would expect to see increasing prevalences of nonvaccine HPV types as prevalences of vaccine HPV types decrease.

Our confirmation of reductions in a cross-protected HPV type is encouraging. However, the results of this systematic review and meta-analysis provide no clear evidence for type replacement because the data are unclear about the extent to which any observed increases result from other temporal changes, changes in the study populations, or an unmasking effect of broad spectrum HPV assays. Large-scale epidemiologic analyses that use various designs have not detected evidence of any interactions between high-risk types, and the known high evolutionary stability of these viruses lessens the risk that type replacement will be a problem (*38**,**39*).

Most women included in the surveillance studies were those vaccinated at older ages (i.e., potentially vaccinated after HPV exposure), and some studies included populations with relatively low coverage, compared with nationally reported vaccination coverage for routine cohorts. Future studies should continue to monitor population-level prevalences of these HPV types. In particular, studies should consider populations vaccinated at young ages and having high vaccination coverage and, perhaps more important, should examine the absolute prevalence of cervical intraepithelial neoplasia 3 lesions attributed to each high-risk HPV type.

Technical Appendix Search strategies and additional details of a meta-analysis of changes in prevalences of nonvaccine human papillomavirus (HPV) types after introduction of HPV vaccination.
