# Supplementary material for: Population-Level Effects of Human Papillomavirus Vaccination Programs on Infections with Nonvaccine Genotypes
Source: Emerg Infect Dis. 2016 Oct;22(10):1732–40. doi: 10.3201/eid2210.160675 (PMC5038419; doi:10.3201/eid2210.160675)
Supplement: Technical Appendix — Search strategies and additional details of a meta-analysis of changes in prevalences of nonvaccine human papillomavirus (HPV) types after introduction of HPV vaccination. [file 16-0675-Techapp-s1.pdf]

Article DOI: http://dx.doi.org/10.3201/eid2210.160675

Population-Level Effects of Human Papillomavirus Vaccination Programs on Infections with Nonvaccine Genotypes

Technical Appendix

Search Details, Study Details, and Prevalence Ratios

Database Search Strategies

Medline Search Strategy: identified 2,410 studies (2016 Feb 19)

1. Epidemiologic Studies/
2. exp case-control Studies/
3. (case* and control*).tw
4. exp Cohort Studies/
5. cohort*.tw
6. Cross-sectional Studies/
7. (cross* and section*).tw
8. Seroepidemiologic Studies/
9. Sentinel Surveillance/
10. Public Health Surveillance/
11. Incidence/
12. Prevalence/
13. Odds Ratio/
14. odds ratio.tw
15. risk ratio.tw
16. rate ratio.tw
17. relative risk.tw

18. screening method.tw

19. effectiveness.tw

20. observational.tw

21. (step* and wedge*).tw

22. Or/1-21

23. Human Papillomavirus DNA Tests/

24. exp Papillomavirus Infections/

25. exp Papillomaviridae/

26. (HPV or papilloma*).tw

27. Uterine Cervical Neoplasms/

28. Genital Neoplasms, Female/

29. Genital Diseases, Female/

30. Uterine Cervical Dysplasia/

31. (Penile ADJ1 wart).tw

32. (cervi* or genit*).tw

33. warts.tw

34. condyloma*.tw

35. neoplas*.tw

36. dysplas*.tw

37. lesion*.tw

38. cancer*.tw

39. carcin*.tw

40. maligna*.tw

41. disease*.tw

42. (carcinoma adj2 situ).tw

43. Or/33-42

44. And/32,43

45. Or/23-30,44

46. (Immunis* or immuniz* or vaccin*).tw

47. Papillomavirus Vaccines/

48. Or/46-47

49. Humans/

50. limit to yr=2007-2016

51. And/22,45,48,49,50

Embase search strategy: identified 3,843 studies (2016 Feb 19)

1. Epidemiology/
2. Cross-sectional study/
3. (cross$ ADJ1 section$).tw
4. exp case control study /
5. (case$ ADJ1 control$).tw
6. cohort analysis/
7. cohort$.tw
8. exp Disease surveillance/
9. exp health survey/
10. incidence/
11. exp prevalence/
12. sentinel surveillance/
13. seroepidemiology/
14. risk/
15. infection risk/
16. population risk/
17. risk reduction/
18. observational study/
19. (odd$ ADJ1 ratio).tw
20. (risk ADJ1 ratio).tw
21. (rate ADJ1 ratio).tw

22. (relative ADJ1 risk).tw

23. (screening ADJ1 method).tw

24. effectiveness.tw

25. observational.tw

26. (step$ ADJ1 wedge$).tw

27. Or/1-26

28. exp Papilloma virus /

29. hpv.tw

30. Papilloma$.tw

31. Uterine cervix disease/

32. Uterine cervix dysplasia/

33. exp Uterine Cervix Tumor/

34. urogenital tract tumor/

35. genital tract tumor/

36. female genital tract tumor/

37. female genital tract cancer/

38. gynecologic cancer/

39. genital tract cancer/

40. female genital tract cancer/

41. Urogenital tract cancer/

42. Female genital tract cancer/

43. female genital tumor/

44. female genital tract infection/

45. genital tract infection/

46. gynecologic infection/

47. (peni$ ADJ1 wart$).tw

48. (cervi$ or genit$).tw

49. wart$.tw

50. condyloma$.tw

51. neoplas$.tw

52. dysplas$.tw

53. lesion$.tw

54. cancer$.tw

55. carcin$.tw

56. maligna$.tw

57. disease$.tw

58. (carcinoma ADJ2 situ).tw

59. Or/49-58

60. And/48,59

61. Or/28-47,60

62. (Immunis$ or immuniz$ or vaccin$).tw

63. Wart virus vaccine/

64. Or/62,63

65. Humans/

66. limit to yr=2007-2016

67. And/27,61,64,65,66

LILACS search strategy: identified 58 studies (2016 Feb 19)

((cross$ AND section$) OR (case$ AND control$) OR (cohort$) OR (odd$ AND ratio) OR (risk AND ratio) OR (rate AND ratio) OR (relative AND risk) OR effectiveness OR observational OR ("step wedge" OR "step-wedge" OR stepwedge)) AND (hpv OR Papilloma$ OR ((cervi$ or genit$) AND (wart$ OR neoplas$ OR dysplas$ OR lesion$ OR cancer$ OR carcin$ OR adeno$ OR squamous$ OR disease$ OR (carcinoma AND situ)))) AND (Immuni$ or vaccin$) AND (PD 2007 OR PD 2008 OR PD 2009 OR PD 2010 OR PD 2011 OR PD 2012 OR PD 2013 OR PD 2014 OR PD 2015 OR PD 2016)

AIM search strategy: identified 17 studies (2016 Feb 19)

hpv OR Papilloma$

References

1. Cameron RL, Kavanagh K, Pan J, Love J, Cuschieri K, Robertson C, et al. Human papillomavirus prevalence and herd immunity after introduction of vaccination program, Scotland, 2009–2013. Emerg Infect Dis. 2016;22:56–64. http://dx.doi.org/10.3201/eid2201.150736

2. Chow EP, Danielewski JA, Fehler G, Tabrizi SN, Law MG, Bradshaw CS, et al. Human papillomavirus in young women with Chlamydia trachomatis infection 7 years after the Australian human papillomavirus vaccination programme: a cross-sectional study. Lancet Infect Dis. 2015;15:1314–23. http://dx.doi.org/10.1016/S1473-3099(15)00055-9

3. Cummings T, Zimet GD, Brown D, Tu W, Yang Z, Fortenberry JD, et al. Reduction of HPV infections through vaccination among at-risk urban adolescents. Vaccine. 2012;30:5496–9. http://dx.doi.org/10.1016/j.vaccine.2012.06.057

4. Kahn JA, Brown DR, Ding L, Widdice LE, Shew ML, Glynn S, et al. Vaccine-type human papillomavirus and evidence of herd protection after vaccine introduction. Pediatrics. 2012;130:e249–56. http://dx.doi.org/10.1542/peds.2011-3587

5. Markowitz LE, Liu G, Hariri S, Steinau M, Dunne EF, Unger ER. Prevalence of HPV after introduction of the vaccination program in the United States. Pediatrics. 2016;137:e20151968. http://dx.doi.org/10.1542/peds.2015-1968

6. Mesher D, Panwar K, Thomas SL, Beddows S, Soldan K. Continuing reductions in HPV 16/18 in a population with high coverage of bivalent HPV vaccination in England: an ongoing cross-sectional study. BMJ Open. 2016;6:e009915. http://dx.doi.org/10.1136/bmjopen-2015-009915

7. Söderlund-Strand A, Uhnoo I, Dillner J. Change in population prevalences of human papillomavirus after initiation of vaccination: the high-throughput HPV monitoring study. Cancer Epidemiol Biomarkers Prev. 2014;23:2757–64. http://dx.doi.org/10.1158/1055-9965.EPI-14-0687

8. Sonnenberg P, Clifton S, Beddows S, Field N, Soldan K, Tanton C, et al. Prevalence, risk factors, and uptake of interventions for sexually transmitted infections in Britain: findings from the National Surveys of Sexual Attitudes and Lifestyles (Natsal). Lancet. 2013;382:1795–806. http://dx.doi.org/10.1016/S0140-6736(13)61947-9

9. Tabrizi SN, Brotherton JM, Kaldor JM, Skinner SR, Liu B, Bateson D, et al. Assessment of herd immunity and cross-protection after a human papillomavirus vaccination programme in Australia: a repeat cross-sectional study. Lancet Infect Dis. 2014;14:958–66. http://dx.doi.org/10.1016/S1473-3099(14)70841-2

Technical Appendix Table 1. Characteristics of studies selected for systematic review and meta-analysis of changes in prevalence of nonvaccine HPV genotypes*

Characteristic	Study (reference no.)								
	Cameron et al. (*1*)	Chow et al. (*2*)	Cummings et al. (*3*)	Kahn et al. (*4*)	Markowitz et al. (*5*)	Mesher et al. (*6*)	Söderlund-Strand et al. (*7*)	Sonnenberg et al. (*8*)	Tabrizi et al. (*9*)
Country of study	Scotland UK	Australia	USA	USA	USA	England, UK	Sweden	Great Britain, UK	Australia
Vaccine introduced	Bivalent	Quadrivalent	Quadrivalent	Quadrivalent	Quadrivalent	Bivalent	Quadrivalent	Bivalent	Quadrivalent
Sample collection, y									
Prevac	2009–2010	2004–2007	1995–2005	2006–2007	2003–2006	2008	2008	1999–2001	2005–2007
Postvac	2011–2013	2007–2014	2010	2009–2010	2009–2012	2010–2013	2012–2013	2010–2012	2010–2011
Specimens tested, no.									
Prevac	2,705	136	150	365	1,795	2,354	11,457	328	202
Postvac	3,010	328	75	383	1,209	7,321	3,555	795	1,058
Study population,† age, y (additional detail)	20–21	≤ 21 (Australian born)	14–17	13–26 (had had sexual intercourse)	14–24	16–25 (sexually active)	All ages	18–44 (sexually experienced)	18–24
Setting for recruiting participants	Cervical screening as part of national cervical screening program	Chlamydia screening at sexual health center in Melbourne, Australia (tested positive)	1 of 3 primary care clinics in Indiana	Hospital-based adolescent clinic and a community health center	Population-based NHANES survey	Chlamydia screening at community sexual health settings	Chlamydia screening in a defined region of Sweden	Households participating in Natsal survey (selected with a stratified probability sample survey)	Cervical screening at sentinel family planning clinics in Sydney, Melbourne, and Perth
Specimen type	Residual LBC	Cervical and high vaginal swab	Self-collected vaginal swab	Cervicovaginal swabs by clinician or self-collected swab	Self-collected cervicovaginal swab	Residual vulval vaginal swab	Genital swabs (alone or immersed in urine)	Urine	Exfoliated cervical cells preserved in PreservCyt‡
Assay for HPV DNA testing	Multimetrix HPV assay	PapType HPV assay	Linear Array HPV Genotyping test	Linear Array HPV Genotyping test	Linear Array HPV Genotyping test	Prevac: Linear Array HPV Genotyping test in those testing positive for Hybrid Capture 2<br><br>Postvac: In-house multiplex PCR- and Luminex-based genotyping system	PCR testing with genotyping by matrix-assisted laser desorption ionization time-of-flight (MALDI-TOF) mass spectrometry	In-house multiplex PCR- and Luminex-based genotyping system	Amplicor DNA test for 13 high-risk types (if negative, tested for presence of mucosal DNA by using L1 consensus primer set PGMY09-PGMY11). If positive for Amplicor or PCMY09/PGMY11, PCR-ELISA were genotyped by using the Linear Array HPV genotyping test
Demographic and sexual behavior data collected	Scottish Index of Multiple Deprivation, mo/y of birth§	Age-stratified PRs were adjusted for by no.male partners, 100%	Samples matched on basis of age at enrollment, clinic site,	Age, race, health care insurance, knowledge of HPV vaccines,	Ethnicity, poverty index, and for those reporting ever having sex, age at first sex,	Age- stratified PRs were adjusted for age, chlamydia positivity at	All samples were anonymised (individual age was known)	Extensive demographic and sexual behavior data collected‡	Age, current use of hormonal contraception, smoking status and postal code

Characteristic	Study (reference no.) Cameron et al. (*1*)	Chow et al. (*2*)	Cummings et al. (*3*)	Kahn et al. (*4*)	Markowitz et al. (*5*)	Mesher et al. (*6*)	Söderlund-Strand et al. (*7*)	Sonnenberg et al. (*8*)	Tabrizi et al. (*9*)
		condom use with all partners in the past 12 mo status, and anatomic sampling method (cervical vs. high vaginal sample)	and reported sexual activity. Data on ethnicity, no. sexual partners in last year and in last 2 mo, no. lifetime sexual partners, no. instances of vaginal intercourse in last year and in the last 2 mo§	smoking status, gynecologic history (no. pregnancies, history of STIs), sexual behaviors (i.e., age at first sex, no. male lifetime partners, no. male partners in last 3 mo, anal sex, condom use)§	lifetime no. partners, no. partners in last 12 mo§	time specimen taken, and collection venue type			of residence§
Vaccination information	Linked from Scottish Immunisation call/recall system and Child Health Schools Programme system	Self-reported; not available for all women	Collected from medical notes	Collected from immunisation registry for 87% of women; collected from self-administered questionnaire for others	Self-reported	Not collected for individuals	Not collected for individuals	Self-reported	Self-reported and validated against the National HPV vaccine register

*Study design for all studies was repeat cross-sectional. HPV, human papillomavirus; LBC, liquid-based cytology; Prevac, prevaccination period; Postvac, postvaccination period; PRs, prevalence ratios.
†Population in all studies were female.
‡Cytyc Corporation, Marlborough, MA, USA.
§These data were not used to adjust the HPV prevalence ratios in this meta-analysis.

Technical Appendix Table 2. Prevalence ratios for nonvaccine high-risk HPV types for female adolescents and women in systemic review and meta-analysis, by age group and vaccine type*

	Bivalent vaccine				Quadrivalent vaccine			
		Heterogeneity				Heterogeneity		
Age group, y/HPV type	No. of studies†	I^2, %	p value	Prevalence ratio (95% CI)	No. studies†	I^2, %	p value	Prevalence ratio (95% CI)
≤19								
Nonavlent vaccine HPV types	2				6			
HPV 31		10.4	0.291	0.54 (0.29–1.03)		8.7	0.36	0.75 (0.60–0.96)
HPV 33		0	0.785	1.66 (0.94–2.92)		0	0.687	0.89 (0.64–1.24)
HPV 45		75.4	0.044	–		0	0.716	1.01 (0.76–1.34)
HPV 52		0	0.408	1.93 (1.34–2.77)		0	0.627	1.20 (0.99–1.47)
HPV 58		0	0.445	1.19 (0.81–1.73)		0	0.742	0.92 (0.69–1.22)
Other high-risk HPV types	2				6			
HPV 35		85.2	0.009	–		0	0.914	0.91 (0.58–1.42)
HPV 39		0	0.755	1.30 (0.89–1.91)		0	0.932	1.26 (1.01–1.58)
HPV 51		74.9	0.046	–		35.2	0.172	1.16 (1.00–1.36)
HPV 56		18.3	0.269	2.08 (1.43–3.04)		64.9	0.014	–
HPV 59		51.9	0.149	–		0	0.478	1.27 (1.03–1.57)
HPV 68		0	0.444	1.84 (0.62–5.47)		0	0.601	1.20 (0.82–1.76)
Other possibly high-risk types	2				4			
HPV 26		0	0.873	1.89 (0.84–4.26)		26.8	0.251	1.21 (0.38–3.81)
HPV 53		0	0.894	2.22 (1.25–3.94)		0	0.445	1.28 (0.88–1.85)
HPV 70		0	0.957	4.07 (1.43–11.55)		0	0.97	0.82 (0.41–1.64)
HPV 73		0	0.926	1.39 (0.98–1.98)		0	0.806	1.32 (0.83–2.07)
HPV 82		0	0.998	2.00 (0.50–7.95)		65.1	0.035	–
20–24								
Nonavalent vaccine HPV types	3				5			
HPV 31		57.8	0.094	–		0	0.889	0.95 (0.81–1.10)
HPV 33		55.0	0.108	–		48.1	0.103	–
HPV 45		74.2	0.021	–		56.9	0.055	–
HPV 52		65.6	0.055	1.26 (0.87–1.83)		0	0.53	1.28 (1.12–1.46)
HPV 58		0	0.499	1.17 (0.94–1.46)		0	0.684	1.12 (0.93–1.34)
Other high-risk HPV types	3				5			
HPV 35		0	0.968	1.22 (0.79–1.87)		43.1	0.134	–
HPV 39		44.8	0.163	1.32 (0.93, 1.88)		0	0.743	1.09 (0.93–1.28)
HPV 51		0	0.57	1.37 (1.16–1.62)		47.0	0.11	1.19 (0.88–1.61)
HPV 56		75.4	0.017	1.45 (0.82–2.59)		87.5	<0.001	–
HPV 59		86.1	0.001	–		0	0.604	1.13 (0.94–1.37)
HPV 68		67.4	0.046	–		0	0.842	0.99 (0.72–1.37)
Other possibly high-risk types	3				3			
HPV 26		69.0	0.04	–		21.1	0.282	1.35 (0.28–6.47)
HPV 53		0.3	0.367	1.23 (1.05–1.45)		16.9	0.3	0.90 (0.64–1.25)
HPV 70		0	0.382	1.11 (0.81–1.51)		0	0.811	2.47 (1.24–4.94)
HPV 73		43.8	0.169	–		76.3	0.015	–
HPV 82		73.7	0.022	–		0	0.989	0.94 (0.39–2.26)

*HPV, human papillomavirus; –, prevalence ratios were not calculated because of heterogeneity of data.
†Number of studies were the same for all HPV types within each category.

Technical Appendix Table 3. Prevalence ratios for nonvaccine high-risk HPV types for female adolescents and women in systemic review and meta-analysis, by age group and potential bias*

	Relatively low potential bias†				Relatively high potential bias‡			
		Heterogeneity				Heterogeneity		
Age group, y/HPV type	No. studies§	I^2,%	p value	Prevalence ratio (95% CI)	No. studies§	I^2,%	p value	Prevalence ratio (95% CI)
≤19								
Nonavalent vaccine HPV types	5				3			
HPV 31		31.2	0.213	–		0	0.447	0.73 (0.58–0.93)
HPV 33		0	0.526	0.79 (0.30–2.06)		34.4	0.218	–
HPV 45		21.5	0.278	0.84 (0.49–1.44)		0.6	0.366	0.99 (0.76–1.31)
HPV 52		0	0.681	1.09 (0.77–1.56)		61.9	0.072	–
HPV 58		0	0.672	0.87 (0.58–1.30)		0	0.505	1.08 (0.82–1.42)
Other high-risk HPV types	5				3			
HPV 35		0	0.424	0.85 (0.46–1.58)		60.6	0.079	–
HPV 39		0	0.907	1.21 (0.83–1.78)		0	0.846	1.30 (1.04–1.61)
HPV 51		45.3	0.120	–		0	0.433	1.28 (1.09–1.50)
HPV 56		69.3	0.011	–		79.9	0.007	–
HPV 59		0	0.465	1.29 (0.94–1.76)		85.9	0.001	–
HPV 68		12.6	0.333	1.21 (0.76–1.93)		0	0.948	1.33 (0.75–2.36)
Other possibly high-risk types	5				1			
HPV 26		3.3	0.388	1.27 (0.45–3.58)		–	–	1.93 (0.82–4.59)
HPV 53		0	0.514	1.32 (0.92–1.90)		–	–	2.19 (1.18–4.04)
HPV 70		0	0.831	0.90 (0.45–1.76)		–	–	4.02 (1.31–12.32)
HPV 73		0	0.909	1.33 (0.87–2.05)		–	–	1.39 (0.96–2.00)
HPV 82		55.0	0.064	–		–	–	2.00 (0.42–9.44)
20–24								
Nonavalent vaccine HPV types	5				3			
HPV 31		27.7	0.237	–		0	0.670	0.95 (0.81–1.11)
HPV 33		0	0.599	0.64 (0.52–0.78)		0	0.424	1.03 (0.83–1.27)
HPV 45		78.5	0.001	–		0	0.948	0.90 (0.74–1.10)
HPV 52		0	0.905	1.06 (0.91–1.22)		11.8	0.322	1.37 (1.20–1.56)
HPV 58		0	0.859	1.04 (0.85–1.28)		0	0.600	1.23 (1.02–1.50)
Other high-risk HPV types	5				3			
HPV 35		0	0.754	1.42 (0.97–2.08)		10.7	0.326	0.90 (0.67–1.21)
HPV 39		8.3	0.359	1.12 (0.94–1.34)		0	0.415	1.14 (0.97–1.34)
HPV 51		32.5	0.205	–		46.9	0.152	–
HPV 56		0	0.914	1.03 (0.89–1.21)		94.5	0.000	–
HPV 59		0	0.443	1.08 (0.91–1.28)		86.4	0.001	–
HPV 68		0	0.692	1.04 (0.72–1.49)		72.5	0.026	–
Other possibly high-risk types	5				1			
HPV 26		54.8	0.065	–		–	–	1.14 (0.37–3.50)
HPV 53		36.3	0.179	–		–	–	1.52 (0.86–2.69)
HPV 70		34.5	0.191	–		–	–	1.64 (0.79–3.37)
HPV 73		56.0	0.059	–		–	–	1.92 (1.04–3.53)
HPV 82		0	0.984	0.75 (0.60–0.94)		–	–	0.22 (0.10–0.51)

*HPV, human papillomavirus; –, prevalence ratios were not calculated because of heterogeneity of data.
†Average-low potential bias includes 6 studies (*1, 3–5, 8, 9*).
‡Average-high potential bias includes 3 studies (*2,6,7*).
§Number of studies were the same for all HPV types within each category.

Technical Appendix Table 4. Prevalence ratio for nonvaccine high-risk HPV types for female adolescents and women in systemic review and meta-analysis, by age group and vaccination coverage*

	Low vaccination coverage (<50%)				High vaccination coverage (≥50%)			
	No.	Heterogeneity		Prevalence ratio	No.	Heterogeneity		Prevalence ratio
Age group, y/ HPV type	studies†	I^2, %	p value	(95% CI)	studies†	I^2, %	p value	(95% CI)
≤19								
Nonavalent HPV types	0				8			
HPV 31		–	–	–		6.4	0.381	0.73 (0.58–0.91)
HPV 33		–	–	–		0	0.471	1.04 (0.78–1.38)
HPV 45		–	–	–		5.5	0.387	0.96 (0.75–1.23)
HPV 52		–	–	–		24.0	0.238	1.34 (1.13–1.59)
HPV 58		–	–	–		0	0.727	1.01 (0.80–1.26)
Other high-risk HPV types	0				8			
HPV 35		–	–	–		25.1	0.229	–
HPV 39		–	–	–		0	0.984	1.27 (1.05–1.54)
HPV 51		–	–	–		43.6	0.088	–
HPV 56		–	–	–		74.3	<0.001	–
HPV 59		–	–	–		66.8	0.004	–
HPV 68		–	–	–		0	0.690	1.26 (0.88–1.81)
Other possibly high-risk types	0				6			
HPV 26		–	–	–		0	0.478	1.63 (0.84–3.16)
HPV 53		–	–	–		3.6	0.394	1.51 (1.10–2.06)
HPV 70		–	–	–		23.6	0.257	1.34 (0.75–2.39)
HPV 73		–	–	–		0	0.961	1.36 (1.03–1.80)
HPV 82		–	–	–		49.0	0.081	–
20–24								
Nonavalent HPV types	5				3			
HPV 31		0	0.838	0.96 (0.83–1.12)		25.5	0.261	–
HPV 33		36.3	0.179	–		0	0.618	0.65 (0.53–0.81)
HPV 45		55.9	0.06	–		62.7	0.068	–
HPV 52		26.1	0.248	–		0	0.513	1.10 (0.94–1.27)
HPV 58		0	0.689	1.21 (1.01–1.45)		0	0.807	1.04 (0.83–1.30)
Other high-risk HPV types	5				3			
HPV 35		30.4	0.219	–		0	0.590	1.29 (0.80–2.07)
HPV 39		5.3	0.377	1.17 (1.00–1.37)		0	0.482	1.08 (0.89–1.30)
HPV 51		56.7	0.056	–		37.8	0.201	–
HPV 56		30.5	0.218	–		91.7	<0.001	–
HPV 59		73.5	0.004	–		0	0.673	1.15 (0.96–1.37)
HPV 68		61.7	0.034	–		0	0.810	1.20 (0.78–1.85)
Other possibly high-risk types	4			–	2			
HPV 26		53.8	0.09	–		0	0.862	1.76 (1.00–3.12)
HPV 53		0	0.522	1.31 (0.95–1.81)		76.6	0.039	–
HPV 70		11.8	0.334	1.72 (1.06–2.79)		0	0.335	1.08 (0.76–1.53)
HPV 73		52.5	0.097	–		0	0.503	1.02 (0.82–1.26)
HPV 82		33.7	0.21	–		0	0.675	0.75 (0.59–0.94)

*HPV, human papillomavirus; –, prevalence ratios were not calculated because of heterogeneity of data.
†Number of studies were the same for all HPV types within each category.

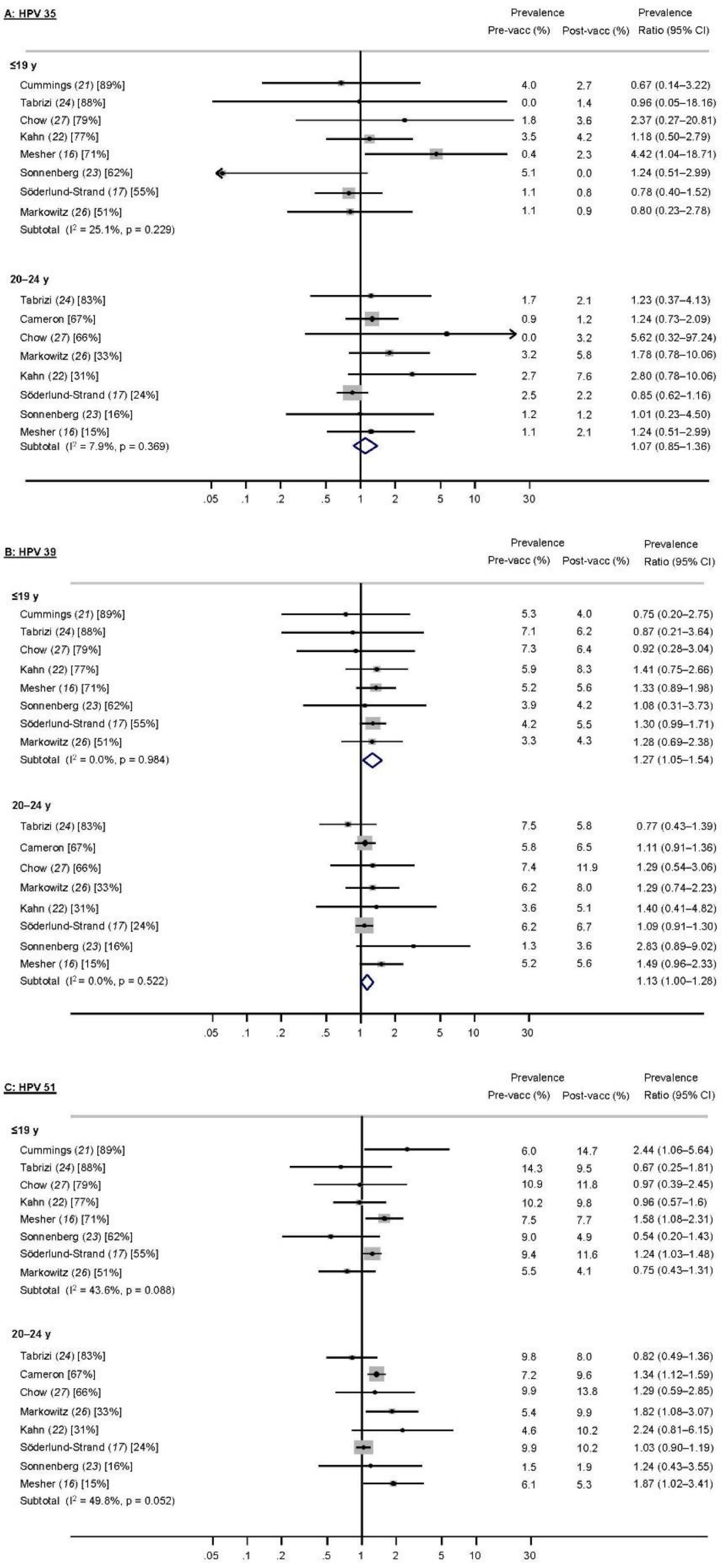

Technical Appendix Figure 1. Prevalence ratios for meta-analysis of changes in other probable high-risk human papillomavirus (HPV) types (HPV35, HPV39, HPV51, HPV56, HPV59, and HPV68) for girls and women, by age group (≤19 and 20–24 years of age). Percentages in square brackets represent vaccination coverage (at least 1 dose) for each study and age group. The size of the dark boxes around the plot points indicates the relative weight given to each study in calculation of the summary estimate. The study by Cameron et al. (*25*) is omitted from analyses for the younger age group because this study included no data for those ≤19 years of age. The study by Cummings et al. (*21)* is omitted from analyses for women 20–24 years of age because this study included no data for this age group. Pre, prevaccination; post, postvaccination.

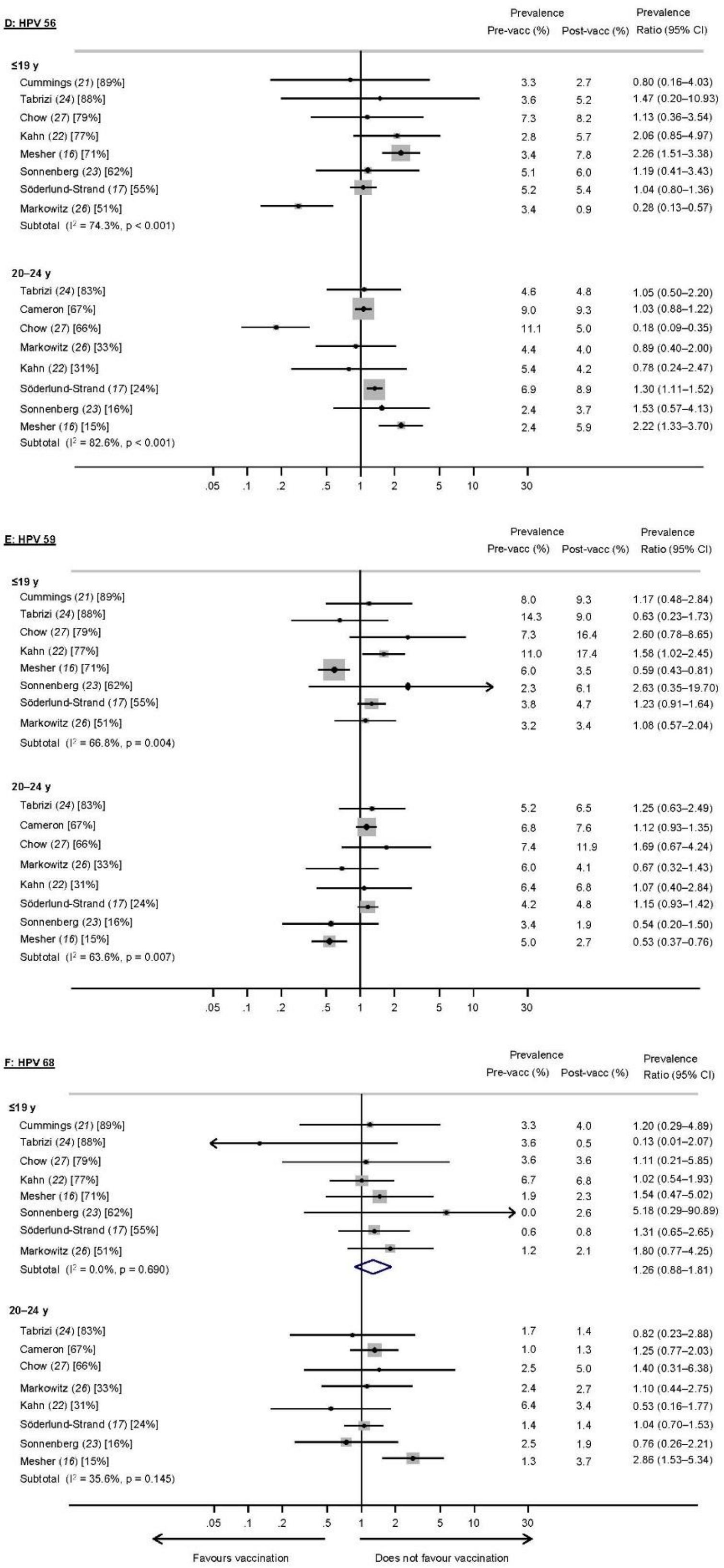
D: HPV 56
Prevalence Pre-vacc (%) Post-vacc (%)
Prevalence Ratio (95% CI)
≤19 y
Cummings (21) [89%] 3.3 2.7 0.80 (0.16–4.03)
Tabrizi (24) [88%] 3.6 5.2 1.47 (0.20–10.93)
Chow (27) [79%] 7.3 8.2 1.13 (0.36–3.54)
Kahn (22) [77%] 2.8 5.7 2.06 (0.85–4.97)
Mesher (16) [71%] 3.4 7.8 2.26 (1.51–3.38)
Sonnenberg (23) [62%] 5.1 6.0 1.19 (0.41–3.43)
Söderlund-Strand (17) [55%] 5.2 5.4 1.04 (0.80–1.36)
Markowitz (26) [51%] 3.4 0.9 0.28 (0.13–0.57)
Subtotal (I² = 74.3%, p < 0.001)
20–24 y
Tabrizi (24) [83%] 4.6 4.8 1.05 (0.50–2.20)
Cameron [67%] 9.0 9.3 1.03 (0.88–1.22)
Chow (27) [66%] 11.1 5.0 0.18 (0.09–0.35)
Markowitz (26) [33%] 4.4 4.0 0.89 (0.40–2.00)
Kahn (22) [31%] 5.4 4.2 0.78 (0.24–2.47)
Söderlund-Strand (17) [24%] 6.9 8.9 1.30 (1.11–1.52)
Sonnenberg (23) [16%] 2.4 3.7 1.53 (0.57–4.13)
Mesher (16) [15%] 2.4 5.9 2.22 (1.33–3.70)
Subtotal (I² = 82.6%, p < 0.001)
.05 .1 .2 .5 1 2 5 10 30
E: HPV 59
Prevalence Pre-vacc (%) Post-vacc (%)
Prevalence Ratio (95% CI)
≤19 y
Cummings (21) [89%] 8.0 9.3 1.17 (0.48–2.84)
Tabrizi (24) [88%] 14.3 9.0 0.63 (0.23–1.73)
Chow (27) [79%] 7.3 16.4 2.60 (0.78–8.65)
Kahn (22) [77%] 11.0 17.4 1.58 (1.02–2.45)
Mesher (16) [71%] 6.0 3.5 0.59 (0.43–0.81)
Sonnenberg (23) [62%] 2.3 6.1 2.63 (0.35–19.70)
Söderlund-Strand (17) [55%] 3.8 4.7 1.23 (0.91–1.64)
Markowitz (26) [51%] 3.2 3.4 1.08 (0.57–2.04)
Subtotal (I² = 66.8%, p = 0.004)
20–24 y
Tabrizi (24) [83%] 5.2 6.5 1.25 (0.63–2.49)
Cameron [67%] 6.8 7.6 1.12 (0.93–1.35)
Chow (27) [66%] 7.4 11.9 1.69 (0.67–4.24)
Markowitz (26) [33%] 6.0 4.1 0.67 (0.32–1.43)
Kahn (22) [31%] 6.4 6.8 1.07 (0.40–2.84)
Söderlund-Strand (17) [24%] 4.2 4.8 1.15 (0.93–1.42)
Sonnenberg (23) [16%] 3.4 1.9 0.54 (0.20–1.50)
Mesher (16) [15%] 5.0 2.7 0.53 (0.37–0.76)
Subtotal (I² = 63.6%, p = 0.007)
.05 .1 .2 .5 1 2 5 10 30
F: HPV 68
Prevalence Pre-vacc (%) Post-vacc (%)
Prevalence Ratio (95% CI)
≤19 y
Cummings (21) [89%] 3.3 4.0 1.20 (0.29–4.89)
Tabrizi (24) [88%] 3.6 0.5 0.13 (0.01–2.07)
Chow (27) [79%] 3.6 3.6 1.11 (0.21–5.85)
Kahn (22) [77%] 6.7 6.8 1.02 (0.54–1.93)
Mesher (16) [71%] 1.9 2.3 1.54 (0.47–5.02)
Sonnenberg (23) [62%] 0.0 2.6 5.18 (0.29–90.89)
Söderlund-Strand (17) [55%] 0.6 0.8 1.31 (0.65–2.65)
Markowitz (26) [51%] 1.2 2.1 1.80 (0.77–4.25)
Subtotal (I² = 0.0%, p = 0.690) 1.26 (0.88–1.81)
20–24 y
Tabrizi (24) [83%] 1.7 1.4 0.82 (0.23–2.88)
Cameron [67%] 1.0 1.3 1.25 (0.77–2.03)
Chow (27) [66%] 2.5 5.0 1.40 (0.31–6.38)
Markowitz (26) [33%] 2.4 2.7 1.10 (0.44–2.75)
Kahn (22) [31%] 6.4 3.4 0.53 (0.16–1.77)
Söderlund-Strand (17) [24%] 1.4 1.4 1.04 (0.70–1.53)
Sonnenberg (23) [16%] 2.5 1.9 0.76 (0.26–2.21)
Mesher (16) [15%] 1.3 3.7 2.86 (1.53–5.34)
Subtotal (I² = 35.6%, p = 0.145)
.05 .1 .2 .5 1 2 5 10 30
Favours vaccination
Does not favour vaccination

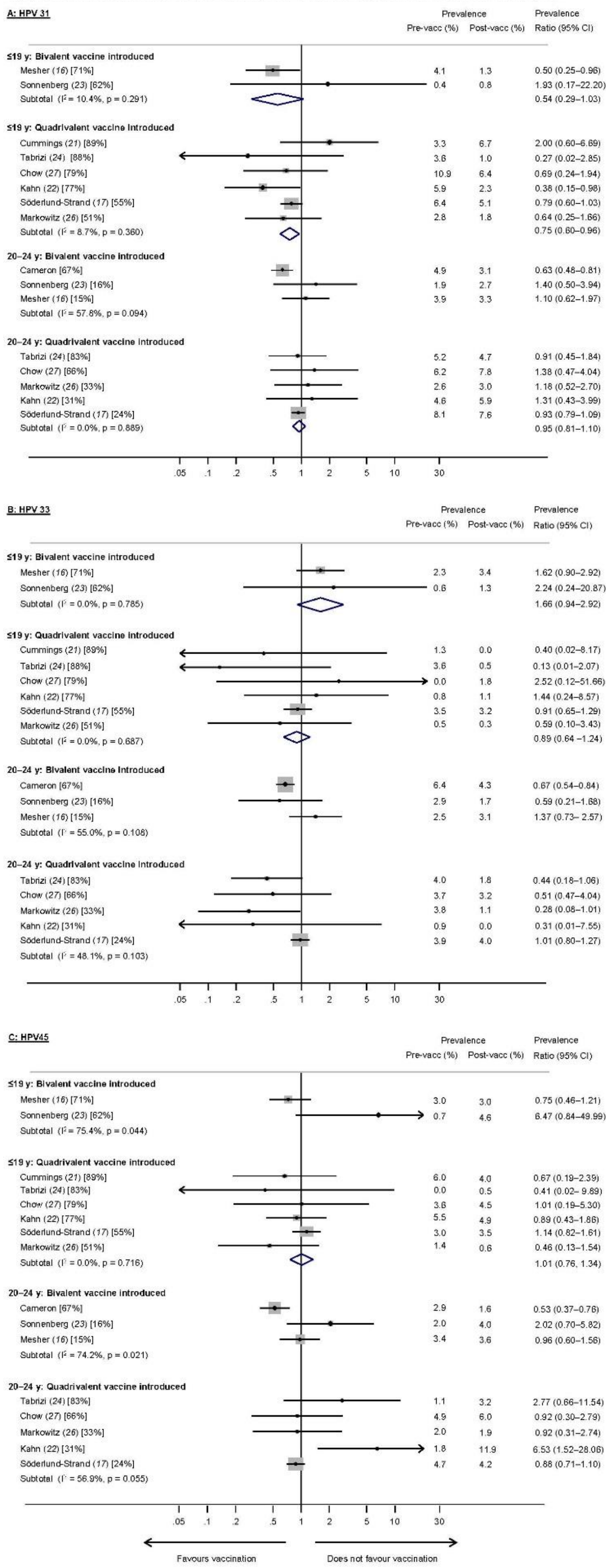

Technical Appendix Figure 2. Prevalence ratios for meta-analysis of changes in high-risk human papillomavirus (HPV) types (HPV31, HPV33, and HPV45) with evidence of cross-protection for girls and women, by age group (<u><</u>19 and 20–24 years of age) and vaccine type. Percentages in square brackets represent vaccination coverage (at least 1 dose) for each study and age group. The size of the dark boxes around the plot points indicates the relative weight given to each study in the calculation of the summary estimate. The study by Cameron et al. (*25*) is omitted from analyses for the younger age group because this study included no data for the group <u><</u>19 years of age. The study by Cummings et al. (*21)* is omitted from analyses for women 20–24 years of age because this study included no data for this age group. Pre, prevaccination; post, postvaccination.

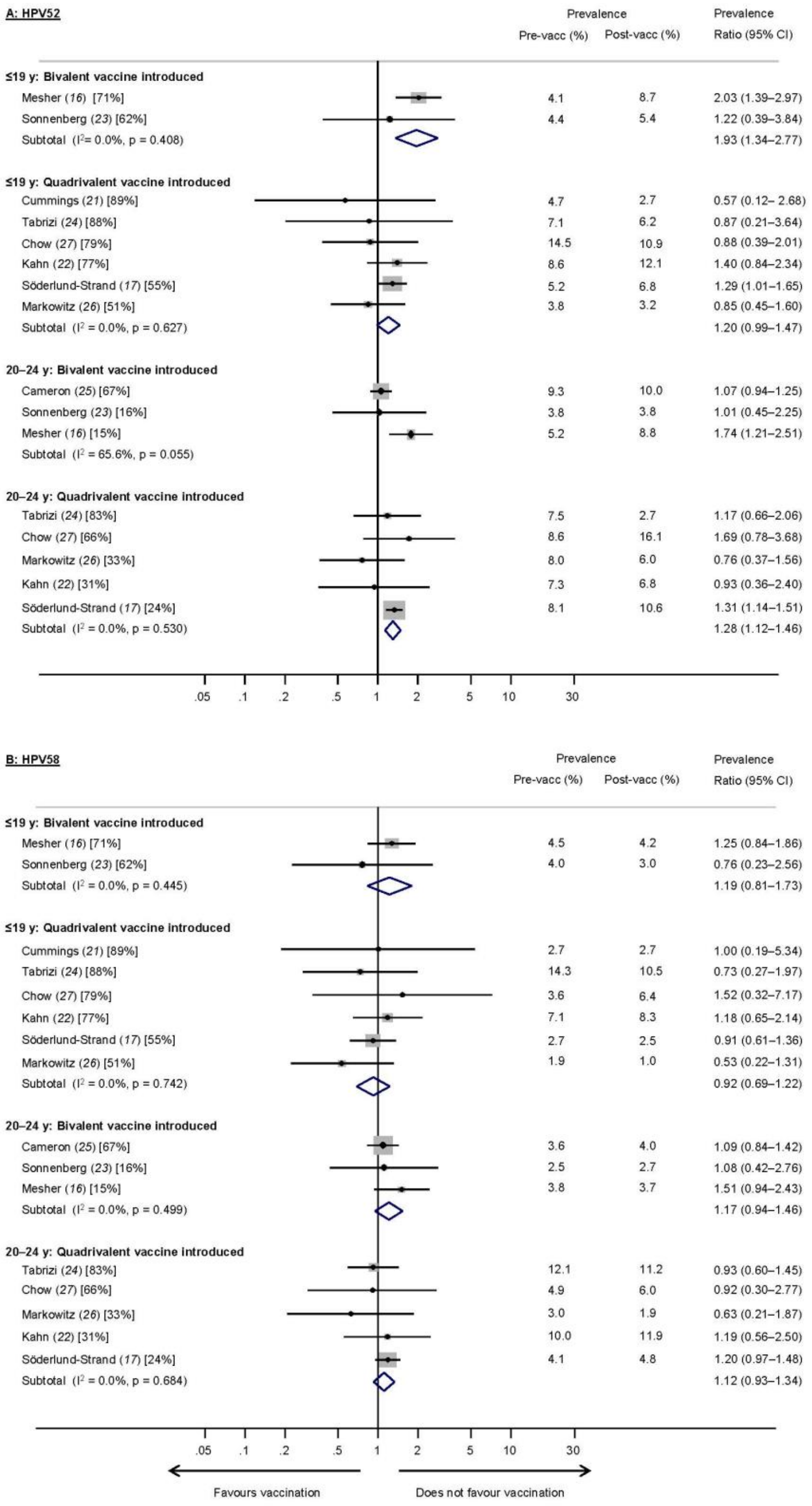

Technical Appendix Figure 3. Prevalence ratios for meta-analysis of changes in other high-risk human papillomavirus (HPV) types(HPV52 and HPV58) included in the nonavalent vaccine for girls and women, by age group (≤19 and 20–24 years of age) and vaccine type. Percentages in square brackets represent vaccination coverage (at least 1 dose) for each study and age group. The size of the dark boxes around the plot points indicates the relative weight given to each study in the calculation of the summary estimate. The study by Cameron et al. (*25*) is omitted from analyses for the younger age group because this study included no data for the group ≤19 years of age. The study by Cummings et al. (*21)* is omitted from analyses for women 20–24 years of age because this study included no data for this age group. Pre, prevaccination; post, postvaccination.

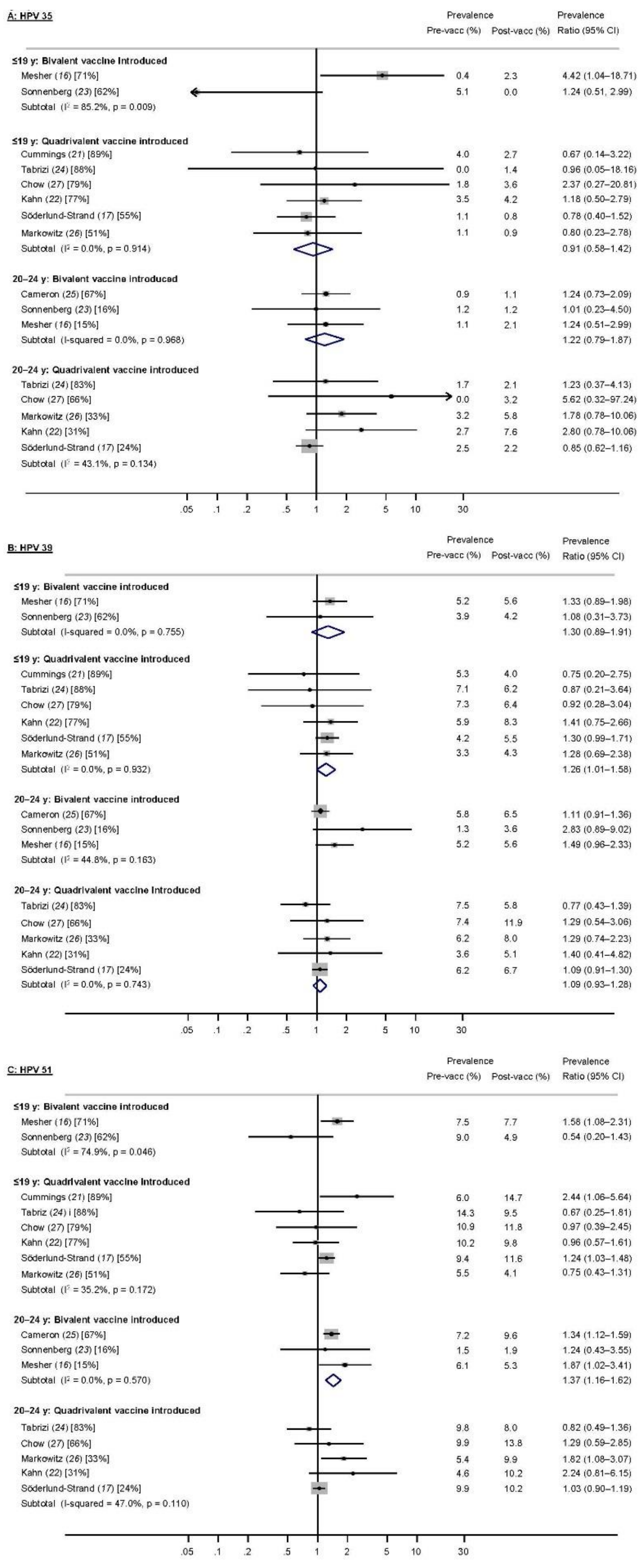

Technical Appendix Figure 4. Prevalence ratios for meta-analysis of changes in other probably high-risk HPV types (HPV35, HPV39, HPV51, HPV56, HPV59, and HPV68) for girls and women, by age-group (<u><</u>19 and 20–24 years of age) and vaccine type. Percentages in square brackets represent vaccination coverage (at least 1 dose) for each study and age group. The size of the dark boxes around the plot points indicates the relative weight given to each study in the calculation of the summary estimate. The study by Cameron et al. (*25*) is omitted from analyses for the younger age group because this study included no data for the group <u><</u>19 years of age. The study by Cummings et al. (*21)* is omitted from analyses for women 20–24 years of age because this study included no data for this age group. Pre, prevaccination; post, postvaccination.

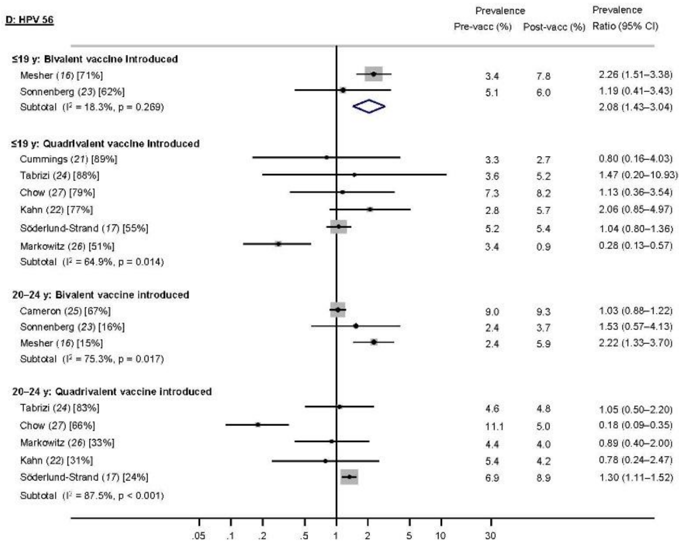
D: HPV 56
Prevalence
Pre-vacc (%) Post-vacc (%)
Prevalence Ratio (95% CI)
≤19 y: Bivalent vaccine introduced
Mesher (16) [71%] 3.4 7.8 2.26 (1.51–3.38)
Sonnenberg (23) [62%] 5.1 6.0 1.19 (0.41–3.43)
Subtotal (I² = 18.3%, p = 0.269) 2.08 (1.43–3.04)
≤19 y: Quadrivalent vaccine introduced
Cummings (21) [89%] 3.3 2.7 0.80 (0.16–4.03)
Tabrizi (24) [88%] 3.6 5.2 1.47 (0.20–10.93)
Chow (27) [79%] 7.3 8.2 1.13 (0.36–3.54)
Kahn (22) [77%] 2.8 5.7 2.06 (0.85–4.97)
Söderlund-Strand (17) [55%] 5.2 5.4 1.04 (0.80–1.36)
Markowitz (26) [51%] 3.4 0.9 0.28 (0.13–0.57)
Subtotal (I² = 64.9%, p = 0.014)
20–24 y: Bivalent vaccine introduced
Cameron (25) [67%] 9.0 9.3 1.03 (0.88–1.22)
Sonnenberg (23) [16%] 2.4 3.7 1.53 (0.57–4.13)
Mesher (16) [15%] 2.4 5.9 2.22 (1.33–3.70)
Subtotal (I² = 75.3%, p = 0.017)
20–24 y: Quadrivalent vaccine introduced
Tabrizi (24) [83%] 4.6 4.8 1.05 (0.50–2.20)
Chow (27) [66%] 11.1 5.0 0.18 (0.09–0.35)
Markowitz (26) [33%] 4.4 4.0 0.89 (0.40–2.00)
Kahn (22) [31%] 5.4 4.2 0.78 (0.24–2.47)
Söderlund-Strand (17) [24%] 6.9 8.9 1.30 (1.11–1.52)
Subtotal (I² = 87.5%, p < 0.001)
.05 .1 .2 .5 1 2 5 10 30

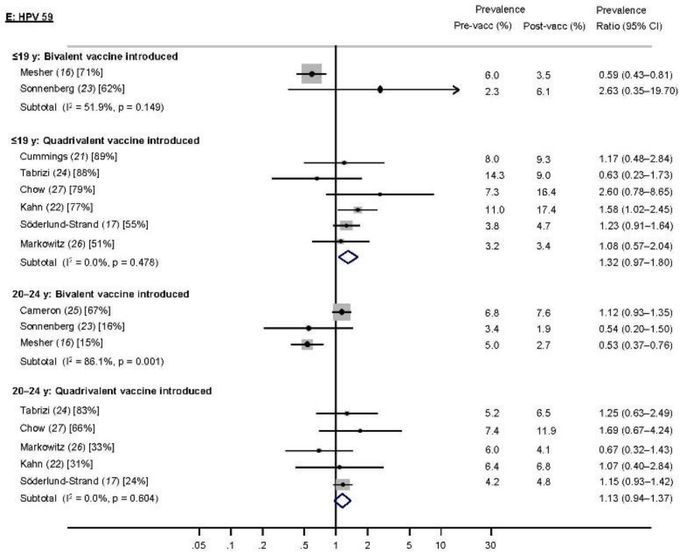
E: HPV 59
Prevalence
Pre-vacc (%) Post-vacc (%)
Prevalence Ratio (95% CI)
≤19 y: Bivalent vaccine introduced
Mesher (16) [71%] 6.0 3.5 0.59 (0.43–0.81)
Sonnenberg (23) [62%] 2.3 6.1 2.63 (0.35–19.70)
Subtotal (I² = 51.9%, p = 0.149)
≤19 y: Quadrivalent vaccine introduced
Cummings (21) [89%] 8.0 9.3 1.17 (0.48–2.84)
Tabrizi (24) [88%] 14.3 9.0 0.63 (0.23–1.73)
Chow (27) [79%] 7.3 16.4 2.60 (0.78–8.65)
Kahn (22) [77%] 11.0 17.4 1.58 (1.02–2.45)
Söderlund-Strand (17) [55%] 3.8 4.7 1.23 (0.91–1.64)
Markowitz (26) [51%] 3.2 3.4 1.08 (0.57–2.04)
Subtotal (I² = 0.0%, p = 0.478) 1.32 (0.97–1.80)
20–24 y: Bivalent vaccine introduced
Cameron (25) [67%] 6.8 7.6 1.12 (0.93–1.35)
Sonnenberg (23) [16%] 3.4 1.9 0.54 (0.20–1.50)
Mesher (16) [15%] 5.0 2.7 0.53 (0.37–0.76)
Subtotal (I² = 86.1%, p = 0.001)
20–24 y: Quadrivalent vaccine introduced
Tabrizi (24) [83%] 5.2 6.5 1.25 (0.63–2.49)
Chow (27) [66%] 7.4 11.9 1.69 (0.67–4.24)
Markowitz (26) [33%] 6.0 4.1 0.67 (0.32–1.43)
Kahn (22) [31%] 6.4 6.8 1.07 (0.40–2.84)
Söderlund-Strand (17) [24%] 4.2 4.8 1.15 (0.93–1.42)
Subtotal (I² = 0.0%, p = 0.604) 1.13 (0.94–1.37)
.05 .1 .2 .5 1 2 5 10 30

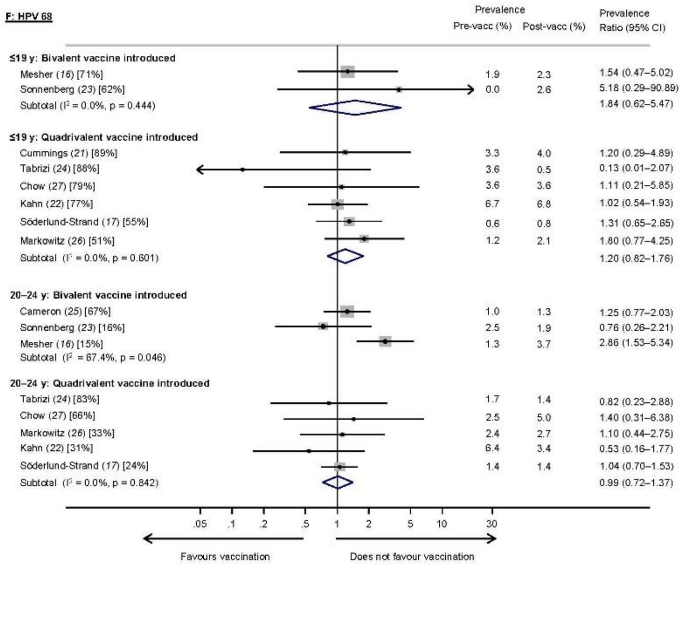
F: HPV 68
Prevalence
Pre-vacc (%) Post-vacc (%)
Prevalence Ratio (95% CI)
≤19 y: Bivalent vaccine introduced
Mesher (16) [71%] 1.9 2.3 1.54 (0.47–5.02)
Sonnenberg (23) [62%] 0.0 2.6 5.18 (0.29–90.89)
Subtotal (I² = 0.0%, p = 0.444) 1.84 (0.62–5.47)
≤19 y: Quadrivalent vaccine introduced
Cummings (21) [89%] 3.3 4.0 1.20 (0.29–4.89)
Tabrizi (24) [88%] 3.6 0.5 0.13 (0.01–2.07)
Chow (27) [79%] 3.6 3.6 1.11 (0.21–5.85)
Kahn (22) [77%] 6.7 6.8 1.02 (0.54–1.93)
Söderlund-Strand (17) [55%] 0.6 0.8 1.31 (0.65–2.65)
Markowitz (26) [51%] 1.2 2.1 1.80 (0.77–4.25)
Subtotal (I² = 0.0%, p = 0.601) 1.20 (0.82–1.76)
20–24 y: Bivalent vaccine introduced
Cameron (25) [67%] 1.0 1.3 1.25 (0.77–2.03)
Sonnenberg (23) [16%] 2.5 1.9 0.76 (0.26–2.21)
Mesher (16) [15%] 1.3 3.7 2.86 (1.53–5.34)
Subtotal (I² = 67.4%, p = 0.046)
20–24 y: Quadrivalent vaccine introduced
Tabrizi (24) [83%] 1.7 1.4 0.82 (0.23–2.88)
Chow (27) [66%] 2.5 5.0 1.40 (0.31–6.38)
Markowitz (26) [33%] 2.4 2.7 1.10 (0.44–2.75)
Kahn (22) [31%] 6.4 3.4 0.53 (0.16–1.77)
Söderlund-Strand (17) [24%] 1.4 1.4 1.04 (0.70–1.53)
Subtotal (I² = 0.0%, p = 0.842) 0.99 (0.72–1.37)
.05 .1 .2 .5 1 2 5 10 30
Favours vaccination
Does not favour vaccination
